# High-Throughput Screening for Epigenetic Compounds That Induce Human β-Defensin 1 Synthesis

**DOI:** 10.3390/antibiotics12020186

**Published:** 2023-01-17

**Authors:** Wentao Lyu, Zhuo Deng, Guolong Zhang

**Affiliations:** 1 State Key Laboratory for Managing Biotic and Chemical Threats to the Quality and Safety of Agro-Products, Institute of Agro-Product Safety and Nutrition, Zhejiang Academy of Agricultural Sciences, Hangzhou 310021, China; 2 Department of Animal and Food Sciences, Oklahoma State University, Stillwater, OK 74078, USA; 3 Center for Excellence in Hip Disorders, Texas Scottish Rite Hospital for Children, Dallas, TX 75219, USA

**Keywords:** antimicrobial peptides, epigenetic compounds, high-throughput screening, histone deacetylase inhibitors, host defense peptides

## Abstract

Antimicrobial host defense peptides (HDPs) are critically important for innate immunity. Small-molecule compounds with the ability to induce HDP synthesis are being actively explored for antimicrobial therapy. To facilitate the discovery of the compounds that specifically activate human β-defensin 1 (*DEFB1*) gene transcription, we established a cell-based high-throughput screening assay that employs HT-29/*DEFB1-luc*, a stable reporter cell line expressing the luciferase gene driven by a 3-Kb *DEFB1* gene promoter. A screening of a library of 148 small-molecule epigenetic compounds led to the identification of 28 hits, with a minimum strictly standardized mean difference of 3.0. Fourteen compounds were further selected and confirmed to be capable of inducing *DEFB1* mRNA expression in human HT-29 colonic epithelial cells. Desirably, the human cathelicidin antimicrobial peptide (*CAMP*) gene was also induced by these epigenetic compounds. Benzamide-containing histone deacetylase inhibitors (HDACi) were among the most potent HDP inducers identified in this study. Additionally, several major genes involved in intestinal barrier function, such as claudin-1, claudin-2, tight junction protein 1, and mucin 2, were differentially regulated by HDP inducers. These findings suggest the potential for the development of benzamide-based HDACi as host-directed antimicrobials for infectious disease control and prevention.

## 1. Introduction

Antimicrobial resistance is a major public health concern worldwide [1]. There is an urgent need for developing novel antimicrobials against drug-resistant pathogens. Host-directed antimicrobial therapy works by targeting the host immune response to a pathogen but not the pathogen directly, and therefore is less likely to trigger resistance [2,3]. Host defense peptides (HDPs), also known as antimicrobial peptides, are an essential component of the innate immune system [4,5]. HDPs are expressed in phagocytic or mucosal epithelial cells, and many are induced in response to infection and inflammation [4,5]. HDPs are capable of killing a broad spectrum of pathogens through electrostatic interactions and subsequent physical disruption of negatively charged membranes, with an extremely low risk of developing resistance [4,5]. In addition, HDPs have a profound impact on modulating innate and adaptive immunity by recruiting and activating different types of immune cells to the site of infection to facilitate pathogen clearance [6]. Enhancing HDP synthesis has emerged as an attractive, host-directed antimicrobial approach for infectious disease control and prevention [7,8].

Mammalian HDPs are composed of two major families, including defensins and cathelicidins [4,5,6]. In humans, defensins consist of three subfamilies known as α-, β-, and θ-defensins, while cathelicidin antimicrobial peptide (CAMP) is the lone member of the cathelicidin family [6]. Human β-defensin 1 (DEFB1) is an important HDP that is constitutively synthesized by skin keratinocytes and mucosal epithelial cells to maintain the health and homeostasis of the intestinal, respiratory, and urogenital tracts [9].

In addition to infection and inflammation, a variety of small-molecule compounds such as butyrate and vitamin D_3_ have the ability to induce HDP synthesis in humans and many other animal species [8,10,11]. Administration of HDP inducers has been found to improve the outcomes of shigellosis, enteropathogenic *E. coli* (EPEC) induced diarrhea, and necrotic enteritis [8,10,11]. Butyrate is a short-chain fatty acid that upregulates HDP gene transcription by acting as a histone deacetylase inhibitor (HDACi) [12,13]. In addition to butyrate, many other structurally unrelated HDACi are also potent HDP inducers [8,14]. HDACi are categorized into five major groups, including benzamides, hydroxamates, cyclic tetrapeptides, aliphatic acids, and sirtuin inhibitors [15]. Classes I, IIa, IIb, and IV HDACi require Zn^2+^ as an essential cofactor, while class III HDACi, also referred to as sirtuins, require NAD^+^ as a cofactor [15,16]. Additionally, other classes of epigenetic compounds such as histone methyltransferase inhibitors (HMTi) and DNA methyltransferase inhibitors (DNMTi) also have the ability to induce HDP synthesis [17]. 

To facilitate the identification of HDP inducers, we and others have developed several different high-throughput screening (HTS) assays [18,19,20,21,22,23], with two assays developed specifically to screen for human *CAMP*-inducing agents [22,23]. However, no HTS assay has been established to allow for the discovery of the compounds that induce human α- or β-defensin genes. In this study, we constructed a stable luciferase reporter cell line under the control of a 3-Kb *DEFB1* gene promoter based on human HT-29 intestinal epithelial cells. Use of the reporter cell line led to identification of 28 hits following HTS of an epigenetic library. The discovery of these novel HDP-inducing epigenetic compounds will accelerate the development of novel host-targeting antimicrobials particularly against drug-resistant infections.

## 2. Results

### 2.1. Development of a Cell-Based HTS Assay

**Figure 1:**
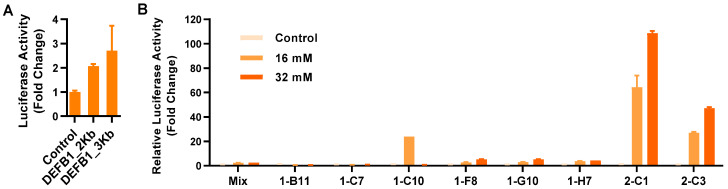
Establishment of stable HT-29/*DEFB1-luc* reporter cell lines. (**A**) Fold changes in the relative luciferase activity of two *DEFB1* gene promoter constructs following transient transfection into HT-29 cells in response to 2 mM sodium butyrate for 24 h. (**B**) Fold changes in the relative luciferase activity of individual clones of stable HT-29/*DEFB1-luc* reporter cells stimulated with or without 16 or 32 mM butyrate for 24 h, relative to the nonstimulated control. The results are means ± SEM of 2–3 independent experiments.

As the first step to develop a stable reporter cell line, two *DEFB1* gene promoter fragments of approximately 2 and 3 Kb were cloned separately into a luciferase reporter vector, pGL-4.21[*luc2P*/Puro] (Promega, Madison, WI, USA). To compare the transcriptional activity of two promoter constructs, a human HT-29 colonic epithelial cell line was transiently transfected with the two recombinant vectors, followed by stimulation with 2 mM sodium butyrate and luciferase assay. Relative to the empty vector, the 2 Kb *DEFB1* gene promoter gave a 2.1-fold increase in luciferase activity, while the 3 Kb construct gave a 2.7-fold increase (Figure 1A). Therefore, the recombinant luciferase reporter vector driven by the 3 Kb gene promoter was chosen for the construction of stable cell lines. Eight stable cell clones were obtained after transfection of HT-29 cells with the recombinant vector, followed by puromycin selection and limiting dilution (Figure 1B). Relative to the other clones, clone 2-C1 was apparently the most sensitive with a 108.6-fold increase in luciferase activity in response to 32 mM butyrate. Therefore, clone 2-C1 was chosen as the HT-29/*DEFB1-luc* stable luciferase reporter cell line for subsequent HTS assays. 

### 2.2. High-Throughput Screening for Epigenetic HDP Inducers

**Figure 2:**
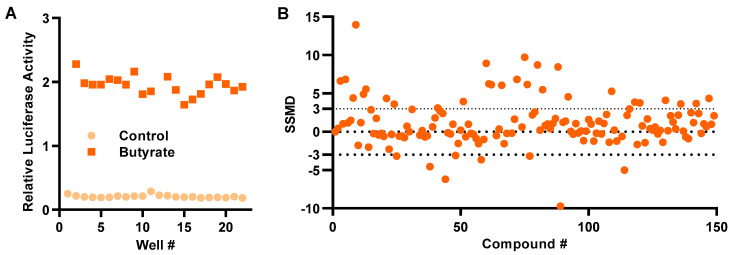
Development of a cell-based high-throughput screening assay to identify HDP-inducing compounds. (**A**) Relative luciferase activity of HT-29/*DEFB1-luc* cells stimulated with or without 16 mM butyrate for 24 h for calculation of Z’-factor. (**B**) Normalized strictly standardized mean difference (SSMD) values of 148 epigenetic compounds following an HTS assay. HT-29/*DEFB1-luc* cells were stimulated with 20 μM of each compound for 24 h, followed by cell viability and luciferase assays. The SSMD value was calculated for each compound from its luciferase activity normalized to the cell viability, as previously described [25]. The dotted lines indicate the threshold for the selection of hits.

To assess the robustness of the HTS assay, HT-29/*DEFB1-luc* cells were stimulated with or without 16 mM butyrate for 24 h in a 96-well plate followed by a luciferase assay. The Z’ factor was calculated to 0.80 ± 0.05 (Figure 2A), indicative of a highly efficient HTS assay [24]. HT-29/*DEFB1-luc* cells were then used to screen an epigenetics screening library of 148 epigenetic compounds (Cayman Chemical, Ann Arbor, MI, USA) at 20 μM. Using the normalized strictly standardized mean difference (SSMD) value of 3.0 as the threshold, which is suggested to be highly stringent [25], 28 hits were identified (Figure 2B). 

### 2.3. Validation of HDP Inducers in HT-29 Cells

**Figure 3:**
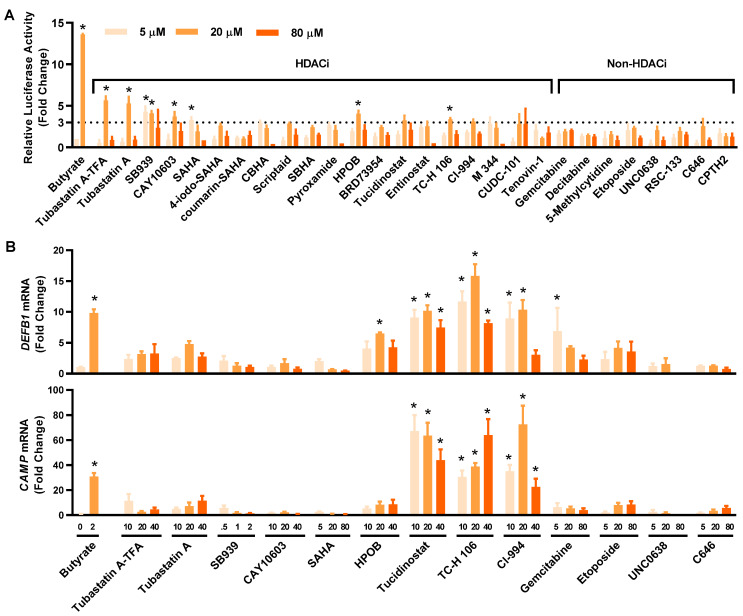
Confirmation of the HDP-inducing activity of the hit compounds. (**A**) Fold changes in the relative luciferase activity of 28 hits in *HT-29/DEFB1-luc* reporter cells stimulated with each compound at 5, 20, and 80 μM for 24 h, followed by the cell viability and luciferase assays. Sodium butyrate (16 mM) was used as a positive control. The hits consisted of 20 HDACi and eight other epigenetic compounds. (**B**) Fold changes in *DEFB1* and *CAMP* mRNA expression levels in response to 14 selected hits. HT-29 cells were stimulated with or without indicated concentrations (µM) of each compound for 24 h, followed by RT-qPCR analysis of gene expression. Sodium butyrate (2 mM) was used as a positive control. The results are means ± SEM of 2–3 independent experiments. Statistical significance was determined by one-way ANOVA and post hoc Dunnett’s test. * *p* < 0.05 compared with the nonstimulated control.

To compare the relative HDP-inducing activity of the hits, HT-29/*DEFB1-luc* cells were treated with each compound at 5, 20, and 80 μM and assayed for luciferase activity. All compounds increased the luciferase activity, with most showing a dose-dependent response (Figure 3A). Among them, tubastatin A and tubastatin A-trifluoroacetate salt (tubastatin A-TFA) were the most potent at 20 μM. Other HDACi, such as SB939, CAY10603, suberoylanilide hydroxamic acid (SAHA), HPOB, tucidinostat, TC-H 106, and CI-994, also increased the luciferase activity by at least three-fold at a minimum of one concentration tested. Although non-HDACi were generally less potent than HDACi, C646, etoposide, UNC0638, and gemcitabine gave a >two-fold change in at least one of three concentrations used. Fourteen compounds were further selected with a >three-fold increase (for an HDACi) or a >two-fold increase (for a non-HDACi) for evaluating their efficacy in inducing HDP gene expression in parental HT-29 cells.

To confirm and compare their ability to induce *DEFB1* mRNA expression, 14 selected hits were applied to HT-29 cells at different concentrations, followed by evaluation of *DEFB1* mRNA expression by reverse transcription-quantitative PCR (RT-qPCR) (Figure 3B, top panel). All HDACi and non-HDACi hits were capable of inducing *DEFB1* mRNA expression. Three HDACi, namely tucidinostat, TC-H 106, and CI-994, were apparently more potent than the other hits. For example, tucidinostat gave an approximately 10-fold increase in *DEFB1* expression at 10 and 20 µM. Among non-HDACi, 5 μM gemcitabine induced *DEFB1* expression by 6.9 fold. The ability of these 14 hits to induce *CAMP* mRNA expression was also evaluated by RT-qPCR (Figure 3B, bottom panel). To our surprise, each compound was also capable of inducing *CAMP* expression. Tucidinostat, TC-H 106, and CI-994 were more potent than others in inducing *CAMP* mRNA expression. Notably, the relative potency of individual compounds had no strong correlations between the HTS and RT-qPCR assays.

### 2.4. Structural and Functional Similarities among Potent HDP Inducers

**Figure 4:**
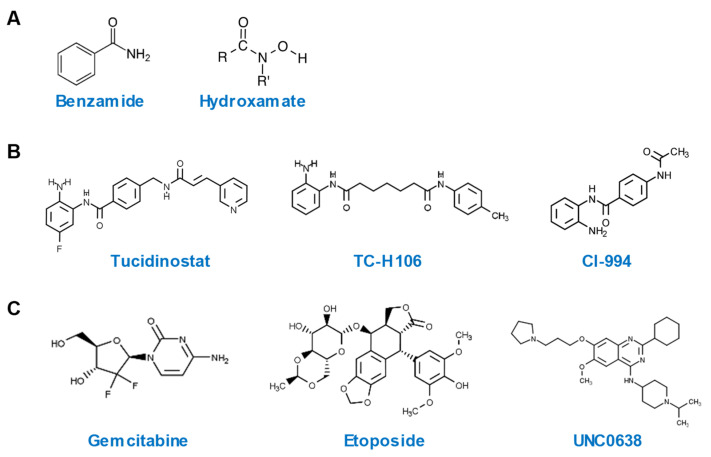
Chemical structures of highly potent HDP-inducing epigenetic compounds. (**A**) Chemical structures of benzamide and hydroxamate. (**B**) Chemical structures of three potent HDP-inducing HDACi. (**C**) Chemical structures of three potent HDP-inducing non-HDACi.

Among a total of 28 hits with an SSMD value of > 3, 20 are HDACi, while the remaining belong to other classes of epigenetic compounds. All HDACi hits are inhibitors of Zn^2+^-dependent HDAC, with the exception of tenovin-1, which is a specific inhibitor of sirtuins [26], a class of NAD^+^-dependent HDAC [27]. All ten most potent HDP-inducing HDACi hits contain a benzamide or hydroxamate structural motif (Figure 4A). It is noteworthy that tucidinostat, TC-H 106, and CI-994, the three HDACi with the most potent ability to induce both human *DEFB1* and *CAMP* genes, are all benzamides (Figure 4B). Potent non-HDACi HDP inducers, on the other hand, are structurally and functionally diverse (Figure 4C). While gemcitabine, decitabine, 5-methylcytidine are nucleoside analogs acting as DNA synthesis inhibitors [28], etoposide is an epipodophyllotoxin and a topoisomerase II inhibitor [29]. UNC0638 and RSC133 function as an HMTi [30] and a DNMTi [31], respectively. C646 and CPTH2 are specific histone acetyltransferase inhibitors (HATi) [32].

### 2.5. Influence of HDP Inducers on Barrier Function

**Figure 5:**
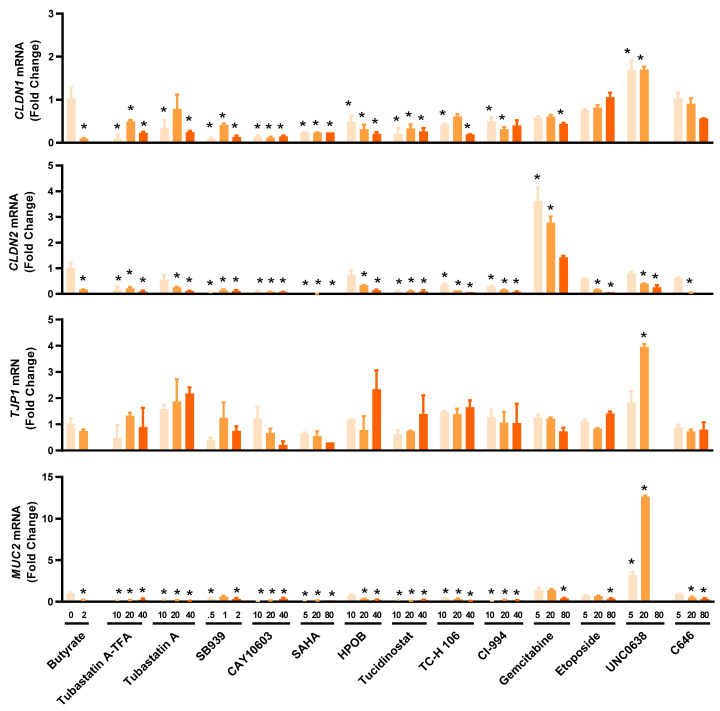
Differential regulation of representative genes involved in intestinal barrier function by selected epigenetic compounds. HT-29 cells were stimulated with or without indicated concentrations (µM) of each compound for 24 h, followed by RT-qPCR analysis of mRNA expression levels of the genes for claudin-1 (*CLDN1*), claudin-2 (*CLDN2*), tight junction protein 1 (*TJP1*), and mucin 2 (*MUC2*). Sodium butyrate (2 mM) was used as a positive control. The results are means ± SEM of 2–3 independent experiments. Statistical significance was determined by one-way ANOVA and post hoc Dunnett’s test. * *p* < 0.05 compared with the nonstimulated control.

Multiple HDP inducers, such as butyrate, vitamin D_3_, lactose, quercetin, and forskolin, have been shown to enhance barrier function [33,34,35,36,37]. To evaluate the potential of newly identified HDP inducers for the control and prevention of mucosal infections, we assessed the expressions of several major genes involved in the formation of tight junctions such as claudin-1 (*CLDN1*), claudin-2 (*CLDN2*), tight junction protein 1 (*TJP1*), and the mucus layer such as mucin 2 (*MUC2*) in HT-29 cells treated with different concentrations of 14 selected HDACi and non-HDACi hits for 24 h (Figure 5). Most HDACi had a negative impact on barrier function by downregulating *CLDN1*, *CLDN2*, and *MUC2* gene expression, although *TJP1* expression was minimally influenced. However, the effect of non-HDACi on barrier function varied greatly among individual compounds. For example, 5 and 20 µM gemcitabine had a negligible influence on *CLDN1*, *TJP1*, and *MUC2* expression, but significantly induced *CLDN2* mRNA. On the other hand, 5 and 20 µM UNC0638 significantly upregulated *CLDN1*, *TJP1*, and *MUC2* mRNA expression, but caused a dose-dependent decrease in *CLDN2* expression.

## 3. Discussion

Human HDPs consist of two major families, including cathelicidins and defensins, that can be induced by infection, inflammation, and a variety of small-molecule compounds [8,10,11]. A number of HDP inducers have been identified, with many regulating HDP gene expression in a species- and/or gene-specific manner. For example, vitamin D_3_ is a potent inducer of the human *CAMP* gene but has no activity to enhance mouse cathelicidin gene expression [38,39]. On the other hand, butyrate universally activates HDP gene expression across multiple animal species [10]. Additionally, not all HDP genes are equally regulated by HDP-inducing compounds because of the presence of different promoter sequences, particularly among different families of HDP genes [13,40]. Although two different HTS assays have been developed for screening for human cathelicidin-inducing compounds [22,23], there is still a need to develop an HTS assay specifically for the discovery of the compounds to induce human defensin genes. 

In this study, we have developed a cell-based HTS assay by employing a stable luciferase reporter cell line, HT-29/*DEFB1-luc*, under the control of a 3 Kb human *DEFB1* gene promoter. The assay is rather robust, with a Z’ factor of 0.80 [24], which is similar to the human *CAMP* HTS assay using a MN8CampLuc reporter cell line that was developed by fusing a luciferase reporter gene with the four-exon open reading frame and a gene promoter segment of human *CAMP* gene [22]. Using a threshold SSMD value of 3.0, a widely accepted standard for hit selection in HTS [25], we have obtained 28 hits (18.9%) out of 148 epigenetic compounds tested. All hits have been subsequently confirmed to dose-dependently increase luciferase activity. A follow-up study has further revealed 14 selected compounds are capable of inducing *DEFB1* mRNA expression, suggesting the validity of our HTS assay. 

However, the relative potency of individual compounds had no strong correlations between the HTS luciferase assay and the RT-qPCR mRNA expression assay. The reason is not completely clear, but it is plausible that *DEFB1* mRNA expression is regulated beyond the 3 Kb gene promoter used in the HTS assay, or additional regulatory elements may be present further upstream of the 3 Kb segment or downstream in the coding or 3′-untranslated regions of the *DEFB1* gene. To our surprise, both human *DEFB1* and *CAMP* genes appear to be regulated similarly by a panel of epigenetic compounds evaluated in this study. It will be interesting to test whether both genes are modulated in a similar fashion by additional epigenetic compounds and non-epigenetic compounds. It is also important to evaluate how other human HDP genes are regulated by these compounds.

Epigenetic modifications of histones and DNA have a profound impact on gene transcription [41,42]. Histone acetylation is a major form of epigenetics that is regulated by two classes of enzymes with opposing activities [43,44]. While HDAC remove acetyl groups from histones, HAT increase the acetylation of histones. HDACi act to suppress the enzyme activity of HDAC. In humans, 11 HDAC have been identified [43,44]. While many HDACi are pan-inhibitors of HDAC, a few are specific for certain individual HDAC [43,44]. In our screening, a majority of the hits are pan-HDACi for Zn^2+^-dependent HDAC. Structurally, they are either benzamides or hydroxamates, consistent with the outcomes of several recent HTS efforts [18,19,21,45], suggesting chromatin relaxation as a result of increased histone acetylation is beneficial for HDP gene activation. In fact, an enhancement of histone H3 acetylation has been shown to positively associate with an increased *DEFB1* gene expression in human lung epithelial cell line A549 [46]. 

Tucidinostat, TC-H 106, and CI-994 are among the most potent inducers of both *DEFB1* and *CAMP* genes identified in this study. Tucidinostat, also known as chidamide, is an HDACi whose structural analogs, such as entinostat [47] and mocetinostat [18], have been identified among the most potent HDP inducers in recent HTS efforts. In fact, entinostat was also identified together with tucidinostat in our initial screening as a hit, with an SSMD value of 3.0; however, it was not selected for further studies because the fold increase was slightly < three at 5, 20 or 80 µM tested in HT-29/*DEFB1-luc* cells (Figure 3A). TC-H 106, also known as pimelic diphenylamide 106, is another HDACi [48] and structurally related to tucidinostat (Figure 4). CI-994, known as tacedinaline or N-acetyldinaline, is another HDACi [49]. These three compounds consist of a benzamide motif and are known to preferentially inhibit class I HDAC, including HDAC1, HDAC2, and HDAC3 [44]. These results are, in fact, in agreement with an earlier study, which revealed the indispensable role of the benzamide motif in *CAMP* gene induction [45]. Therefore, benzamide-containing compounds may be explored further for their HDP-inducing activity and, more importantly, for their efficacy in antimicrobial therapy.

Besides HDACi, we have also identified several non-HDACi with strong HDP-inducing activity. UNC0638 is an HMTi [30], while RSC-133 is a DNMTi [31]. Both HMTi and DNMTi are known to be weak inducers of HDP genes, and both are capable of synergizing with HDACi in HDP gene expression [17]. Four DNA synthesis inhibitors, including gemcitabine, decitabine, 5-methylcytidine, and etoposide, have also been identified. It is unknown how inhibition of DNA synthesis may facilitate HDP gene transcription. To our surprise, C646 and CPTH2, two specific HATi [32], have also been found to induce *DEFB1* gene expression. In contrast to HDACi, HATi suppress histone acetylation, resulting in chromatin condensation and reduced gene transcription [44]. It is paradoxical that both HDACi and HATi, two classes of epigenetic compounds with opposing activity in histone acetylation, are capable of HDP gene induction. Further investigation on the influence of the histone acetylation status of HDP gene transcription is warranted. Fine-tuning on the balance between histone acetylation and deacetylation may have a dramatic impact on regulating HDP gene expression. 

Notably, a majority of epigenetic compounds are being developed as anticancer drugs [16]. It will be interesting to test whether increased HDP synthesis constitutes an anticancer mechanism of epigenetic compounds, besides host defense. In agreement with this hypothesis, HDPs have been reported to selectively induce apoptosis and necrosis of cancer cells through membrane disruption [50,51]. It is perhaps not surprising that, although the compounds are used in HT-29 cells at subtoxic concentrations generally with < 20% cytotoxicity, a majority have a negative impact on barrier function. Therefore, the dose of these HDP-inducing compounds used in antimicrobial therapy needs to be fine-tuned in order to achieve an optimal outcome. However, a few other HDP-inducing compounds such as UNC0638, an HMTi, appear to enhance barrier function (Figure 5). In fact, several other HMTi such as BIX01294 and UNC1999 have also been reported to have barrier-protective properties [17]. Additionally, HDP inducers, such as butyrate, vitamin D_3_, lactose, quercetin, and forskolin, are capable of enhancing barrier integrity as well [33,34,35,36,37]. Therefore, it is not mutually exclusive for a compound to possess both HDP-inducing and barrier-protective activities. It is perhaps more desirable to use those compounds with a dual beneficial effect for antimicrobial therapy of enteric and respiratory infections.

## 4. Materials and Methods

### 4.1. Construction of a Luciferase Reporter Vector Driven by the Human DEFB1 Gene Promoter 

Human genomic DNA was isolated from a human colonic epithelial cell line, HT-29, using Quick-gDNA microPrep (Zymo Research, Irvine, CA, USA) for subsequent amplification of the *DEFB1* gene promoter using Advantage 2 PCR Kit (Takara Bio USA, Mountain View, CA, USA). Two different forward primers (5′-TGG CCT AAC TGG CCG GTA CCA TTC TGA GCA AAC TAT C-3′, and 5′-TGG CCT AAC TGG CCG GTA CCG CTG GTC TCG AAC TCC TAA CCT-3′) were paired with a common reverse primer (5′-CCG GAT TGC CAA GCT TGC AGG CAA CAC CCA GGA TTT C-3′) for cloning approximately 2 and 3 Kb *DEFB1* gene promoter segments upstream of the start codon, respectively. Both forward primers contained a *Kpn*1 site as underlined, while the reverse primer included a *Hind*III site. PCR products were cloned into linearized pGL-4.21[*luc2P*/Puro] Luciferase Reporter Vector (Promega, Madison, WI, USA) using In-Fusion Cloning Kit (Takara Bio USA) following the manufacturer’s instructions. The presence of gene-specific sequences in the recombinant plasmids was confirmed by Sanger sequencing.

### 4.2. Transient Transfection and Luciferase Assay

HT-29 cells were seeded at 5 × 10^4^ cells/well overnight in a 24-well plate at 5% CO_2_ and 37 °C in RPMI 1640 medium (Lonza, Allendale, NJ, USA) supplemented with 10% fetal bovine serum (FBS) (Atlanta Biologicals, Lawrenceville, GA, USA) and 1% penicillin/streptomycin (Invitrogen, Carlsbad, CA, USA). Cells were transfected separately with 50 ng/well of two different *DEFB1* gene promoter-driven luciferase reporter plasmids and 0.15 µL FuGENE HD Transfection Reagent (Promega). After 24 h, cells were stimulated with or without 2 mM sodium butyrate for another 24 h. The luciferase activity was measured using Steady-Glo Luciferase Assay System (Promega, Madison, WI, USA) and read on Modulus Single-Tube Luminometer (Turner Biosystems, Sunnyvale, CA, USA).

### 4.3. Establishment of a Stable Luciferase Reporter Cell Line

To establish a stable luciferase reporter cell line, 750 ng/well of *Kpn*I-linearized 3 Kb *DEFB1* luciferase reporter plasmid was transfected into HT-29 cells in a 6-well tissue culture plate using 2.25 µL/well of FuGene HD (Promega). After 48 h incubation, cells were replenished with fresh complete RPMI 1640 medium supplemented with 1 μg/mL puromycin. The cell culture medium was changed every 2–3 days. After one week of puromycin selection, cells were expanded into a 10 cm cell culture dish and cultured for another week in the presence of 1 µg/mL puromycin, with medium change every 2–3 days. Single cell clones were obtained by subsequent limiting dilution of the cells at 0.1 and 1 cell/well in 96-well plates in complete RPMI 1640 medium containing 1 µg/mL puromycin. After 10–14 days, individual cell clones were gradually expanded and assessed for their responsiveness to sodium butyrate. The most responsive stable cell clone, named HT-29/*DEFB1-luc*, was maintained in complete RPMI 1640 medium supplemented with 1 μg/mL puromycin and subcultured every 3–4 days.

### 4.4. Assessment of Z’-Factor of the High-Throughput Screening (HTS) Assay

HT-29/*DEFB1-luc* cells were seeded at 2 × 10^4^ cells/well overnight in 50 μL complete RPMI 1640 medium in a white 96-well tissue culture plate with clear bottom (Santa Cruz Biotechnology, Dallas, TX, USA), and stimulated with or without 16 mM sodium butyrate for 24 h followed by luciferase assay using Steady-Glo Luciferase Assay System (Promega, Madison, WI, USA) and L-Max II Luminescence Microplate Reader (Molecular Devices, Sunnyvale, CA, USA). Z’ factor was calculated as previously described [24].

### 4.5. High-Throughput Screening of an Epigenetics Compound Library

An epigenetics screening library was purchased from Cayman Chemical (Ann Arbor, MI, USA). The library consists of 148 small-molecule epigenetic compounds that are known to modulate the activity of a variety of epigenetic ‘writers and erasers’ and ‘reader’ proteins such as DNA/histone methyltransferases, DNA/histone demethylases, histone acetyltransferases, histone deacetylases, and acetylated histone binding proteins. All compounds were provided as 10 mM stocks in dimethyl sulfoxide (DMSO). An aliquot of each compound was further diluted to 0.1 mM in serum-free RPMI 1640 medium. HT-29/*DEFB1-luc* cells were seeded at 2 × 10^4^ cells/well in 50 μL of complete RPMI 1640 medium in white 96-well plates overnight and treated with individual epigenetic compounds at the final concentration of 20 μM for 24 h, followed by luciferase assay. To measure cell viability, 5 μL alamarBlue Reagent (Thermo Fisher Scientific, Waltham, MA, USA) was added to individual wells and incubated for 4 h prior to cell lysis and luciferase assay. The cell viability was measured on FLx80 Microplate Fluorescence Reader (BioTek Instruments, Winooski, VT, USA) at 545 nm excitation and 590 nm emission, followed by luciferase assay on L-Max II Luminescence Microplate Reader (Molecular Devices). The luciferase activity of each compound was normalized to its cell viability. The strictly standardized mean difference (SSMD) [25] was calculated for each compound, and the positive hits were identified with a normalized SSMD value of no less than 3.0 [25].

### 4.6. Validation of the Hit Compounds

All hit compounds were purchased from Cayman Chemical (Ann Arbor, MI, USA) and dissolved to 10 mM in DMSO for further validation of their HDP-inducing activity. An aliquot of each compound was further diluted in serum-free RPMI 1640 medium before being applied to cells. HT-29/*DEFB1-luc* cells were seeded at 2 × 10^4^ cells/well in 96-well plates overnight prior to stimulation with each compound at 5, 20, and 80 μM for 24 h prior to luciferase assay. The final concentration of DMSO in cell culture never exceeded 0.5%, which had no influence on cell viability or HDP gene expression. The compounds with a fold change of no less than 3.0 were assessed for their ability to induce representative HDP and tight junction genes in parental HT-29 cells, as described below. 

### 4.7. RNA Extraction and Reverse Transcription-Quantitative PCR (RT-qPCR)

HT-29 cells were stimulated with individual compounds at 5, 20, and 80 μM for 24 h in 12-well tissue culture plates, followed by total RNA isolation using RNAzol^®^ RT Reagent (Molecular Research Center, Cincinnati, OH, USA). For cDNA synthesis, 0.3 μg RNA was used in 4-μL reactions using iScript cDNA Synthesis Kit (Bio-Rad Laboratories, Hercules, CA, USA). PCR was performed in 10-μL reactions using Maxima SYBR Green qPCR Master Mix (Thermo Fisher Scientific, Waltham, MA, USA) on iQ5 Real Time PCR Detection System (Bio-Rad Laboratories). Gene-specific primers for human HDP genes including *DEFB1* and *CAMP* as well as the major genes involved in barrier function such as claudin-1 (*CLDN1*), claudin-2 (*CLDN2*), tight junction protein 1 (*TJP1*), and mucin 2 (*MUC2*) and a reference gene β-actin were previously described [35,37]. Fold change for each gene was calculated with the ΔΔCt method normalized to the β-actin expression levels as described [35,37].

### 4.8. Data Analysis

GraphPad Prism (San Diego, CA, USA) was used for statistical analysis and data visualization. All results were presented as means ± standard error of the mean (SEM) and compared with the nonstimulated control using one-way analysis of variance (ANOVA) and post hoc Dunnett’s test. Statistical significance was considered if *p* < 0.05.

## Data Availability

All data generated during this study are included in this published article.

## Abbreviations

ANOVA: analysis of variance; CAMP, cathelicidin antimicrobial peptide; CLDN1, claudin-1; CLDN2, claudin-2; DEFB1, human β-defensin 1; DMSO, dimethyl sulfoxide; DNMT, DNA methyltransferase; DNMTi, DNMT inhibitor; HAT, histone acetyltransferase; HATi, HAT inhibitor; HDAC, histone deacetylase; HDACi, HDAC inhibitor; HDPs, host defense peptides; HMT, histone methyltransferase; HMTi, HMT inhibitor; MUC2, mucin 2; RT-qPCR, reverse transcription-quantitative PCR; SEM, standard error of the mean; SSMD, strictly standardized mean difference; TJP1, tight junction protein 1.
